# New Insights in Pleural Mesothelioma Classification Update: Diagnostic Traps and Prognostic Implications

**DOI:** 10.3390/diagnostics12122905

**Published:** 2022-11-22

**Authors:** Maria Giovanna Mastromarino, Alessandra Lenzini, Vittorio Aprile, Greta Alì, Diana Bacchin, Stylianos Korasidis, Marcello Carlo Ambrogi, Marco Lucchi

**Affiliations:** 1 Department of Cardiology, Thoracic and Vascular Medicine, Division of Thoracic Surgery, Pisa University Hospital, 56124 Pisa, Italy; 2 Anatomic Pathology Section, Department of Surgical, Medical, Molecular Pathology and Critical Area, University di Pisa, 56124 Pisa, Italy

**Keywords:** malignant pleural mesothelioma, tumor grade, mesothelioma classification, histological subtypes, epithelioid mesothelioma, histology, mesothelioma diagnosis

## Abstract

The 2021 WHO Classification of Tumors of the Pleura has introduced significant changes in mesothelioma codification beyond the three current histological subtypes—epithelioid, sarcomatoid and biphasic. Major advances since the 2015 WHO classification include nuclear grading and the introduction of architectural patterns, cytological and stromal features for epithelioid diffuse mesothelioma. Mesothelioma in situ has been recognized as a diagnostic category. Demonstration of loss of BAP1 or MTAP by immunohistochemistry, or CDKN2A homozygous deletion by FISH, is valuable in establishing the diagnosis of epithelioid mesothelioma. Recent emerging data proved that grading and histological subtypes have prognostic implications and may be helpful to patient risk stratification and clinical management. Nevertheless, the latest mesothelioma classification increases the already non-negligible diagnostic pitfalls, especially concerning differential diagnosis of pre-invasive tumors. In this review, recent changes in histologic classification of mesothelioma and advances in molecular markers are presented and their relation to diagnostic challenges and prognostic implications is discussed.

## 1. Introduction

Malignant pleural mesothelioma (MPM) is a challenging rare neoplasm associated with a dismal prognosis. Despite recent molecular and immunologic breakthroughs deeply renewing the management of thoracic cancer, MPM still remains a deadly disease, with a median survival of 8 to 12 months if untreated and a 5-year survival rate of 5% [1].

The 2015 WHO classification of MPM recognized three major histological subtypes—namely epithelioid, biphasic and sarcomatoid—with different implications on prognosis as well as on treatment strategy [2]. It is well known that epithelioid MPM is associated with better prognosis when compared to biphasic and sarcomatoid subtypes [2].

However, in the last decade, emerging biological data has supported the need for a more exhaustive and clinically valid classification beyond the three current subtypes.

The epithelioid MPM is associated with a more encouraging behavior as well as a remarkable morphological heterogeneity. The role of different architectural patterns, cytologic features and stromal characteristics has recently been reported [3,4]. These additional factors could be helpful to further stratify prognosis and to identify patients who can benefit from multimodal treatment.

Grading has not been previously recommended for mesotheliomas, even though the recent literature has demonstrated the prognostic influence of adjunctive morphological features such as nuclear grade atypia, mitotic count and necrosis, which may pave the way to a more complete risk stratification of patients in regard to clinical management [5,6,7,8].

Therefore, on the thrust of a critical review of the traditional histologic classification of MPM, sponsored by the European Network for Rare Adult Solid Cancers (EURACAN) and the International Association for the Study of Lung Cancer (IASCL) [9], the 2021 (fifth edition) WHO Classification of Thoracic Tumors has recently been published [10].

Critical issues raised by the above-mentioned international multidisciplinary group included the need to update the current classification system to include architectural patterns, stromal and cytologic features; the molecular landscape of MPM; image-acquisition protocols; surgical sample details; and proposals for improving research investigation and clinical trials [9].

Substantial changes in the 2021 WHO Classification of Tumors of the Pleura since the previous classification system include the following [10]: (a) well-differentiated papillary mesothelioma (WDPM) has been renamed as well-differentiated papillary mesothelial tumor (WDPMT), given growing evidence that these tumors exhibit relatively indolent behavior [11,12]; (b) mesothelioma in situ (MIS) has been recognized as a precisely defined clinico-pathologic entity and for the first time included in the 2021 WHO classification. Demonstration of loss of BAP1 and/or MTAP by immunohistochemistry (IHC) and/or CDKN2A (p16) homozygous deletion by fluorescence in situ hybridization (FISH) are essential in differential diagnosis from reactive mesothelial proliferation [13,14,15,16]; (c) the prefix “malignant” has been deleted from localized and diffuse mesothelioma because all mesotheliomas are considered as malignant now that WDPMT and MIS are included in the group of benign or preinvasive mesothelial tumors; (d) the three main histologic subtypes (namely epithelioid, biphasic, and sarcomatoid) remain the same, but architectural patterns and cytologic and stromal features have been formally incorporated into the 2021 classification on the basis of their prognostic implication; (e) nuclear grading for epithelioid diffuse mesothelioma has been introduced for the first time.

Currently, no grading for sarcomatoid or biphasic mesotheliomas is recommended, and diagnostic pathologic criteria remain unchanged, except for the diagnosis of biphasic mesothelioma in small biopsies, in which either epithelioid or sarcomatoid components no longer require a minimum of 10% [10]. The major advances in the 2021 WHO classification of pleural mesothelioma is summarized in Table 1.

The purpose of applying a grading system to epithelioid diffuse mesothelioma would be advantageous to identify tumors characterized by a more aggressive behavior. Moreover, patients affected by epithelioid subtype are those who would benefit the most from improved risk stratification concerning clinical management.

This review aims to provide an extensive insight into the pathologic update of pleural mesothelioma classification, in order to investigate the prognostic relevance of the latest upgrades and their implications on potentially diagnostic pitfalls.

## 2. Materials and Methods

A comprehensive literature research was performed using the Pubmed/Medline electronic databases to identify all relevant data on the histopathological classification of pleural mesothelioma.

Key words used were combination of “malignant pleural mesothelioma”, “grading”, “histological subtypes”, “pleural mesothelioma classification” and “epithelioid mesothelioma”.

The search was limited to the English language and relevant studies published from 2012 to May 2022 were identified, screened and reviewed by all the authors. WHO guidelines were also included and referenced.

We conducted accurate research of all studies focused on advances in the molecular understanding of pleural mesothelioma, histologic features and diagnostic criteria incorporated in the new 2021 WHO classification update.

Papers focusing on extra-thoracic malignant mesothelioma, unpublished material, congress abstracts, letters, editorials and case reports were excluded.

**Figure 1:**
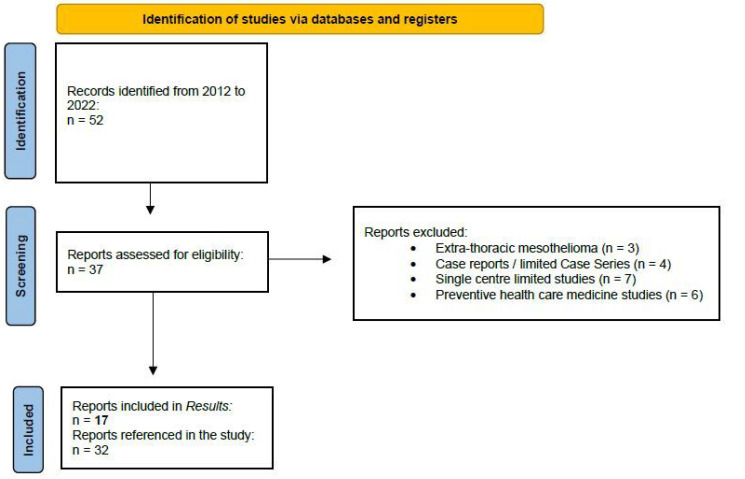
The flow diagram showing the criteria for the identification and selection of the studies included in this review.

The research method for the identification of the studies included in this review is shown in Figure 1.

## 3. Results

### 3.1. Histologic Characteristics and Grading of Pleural Diffuse Epithelioid Mesothelioma (Table 2)

Kadota et al. firstly highlighted the prognostic impact of nuclear features as concerns epithelioid MPM in 2012 [5]. The authors collected the clinical and pathological data of 232 patients affected by epithelioid MPM from a single institution, the Memorial Sloan-Kattering Cancer Center, between 1989 and 2009. Seven nuclear features were considered: nuclear atypia, nuclear/cytoplasmic ratio, chromatin pattern, intranuclear inclusions, prominence of nucleoli, mitotic count and atypical mitoses. Nuclear atypia (*p* = 0.012) and mitotic count (*p* < 0.001) proved independent prognostic factors on multivariate analysis, and these two factors were utilized to create a three-tier nuclear grade score. For nuclear atypia, tumors were scored as 1 for mild, 2 for moderate, and 3 for severe atypia. For mitotic count, tumors were scored as 1 for low, 2 for intermediate, and 3 for high. A total score was computed as the sum of the two-parameter scores, ranging from 2 to 6, moving from the best to the worst overall survival (OS). The resulting nuclear grade stratified patients into three distinct prognostic groups: grade I, for total scores of 2 or 3 (*n* = 107, median OS = 28 months), grade II, for total scores of 4 or 5 (*n* = 91, 14 months) and grade III, for total scores of 6 (*n* = 34, 5 months). Nuclear grade was revealed not only as an independent predictor of OS (*p* < 0.001), but it was also a stronger discriminator of survival than all currently available factors. Furthermore, nuclear grade was associated with time to recurrence (*p* = 0.004) in patients who underwent complete surgical resection (*n* = 159).

Similarly, Pelosi et al. proposed a multicenter, retrospective study involving 940 patients affected by MPM (328 in a training set and 612 in a validation set), with a diagnosis based on either biopsy or resection specimens between October 1980 and June 2015 [6]. The authors presented a Pathologic Grading System (PGS), created by attributing to each histologic parameter which resulted as independent on multivariate analysis, different scores based on the increase in corresponding hazard ratios. The PGS was constructed by taking into account the histological subtyping of MPM (epithelioid/biphasic = 0 points; sarcomatoid = 2 points), necrosis (absent = 0 points versus present = 1 point), mitotic count per 1 mm2 (cutoffs as follows: 1–2 = 0 points, 3–5 = 1 point, 6–9 = 2 points, or ≥10 = 4 points), and the Ki-67 labeling index (LI) based on 2000 cells (<30% = 0 points versus ≥30% = 1 point). The relevant PGS score thus ranged from 0 to 8 points for individual patients with MPM. Histological variants and type of surgical samples (i.e., biopsies or resection specimens) showed no correlation with survival outcomes, whereas the PGS outperformed mitotic count and Ki-67 LI in both the training (AUC = 0.76) and validation sets (AUC = 0.73), *p* < 0.01. Patient survival progressively deteriorated from a score of 0 (median times of 26.3 and 26.9 months) to a score of 1–3 (median times of 12.8 and 14.4 months) and a score of 4–8 (median times of 3.7 and 7.7 months) in both groups. The training set showed a 46% increase in risk of death per 1-point increase in PGS score (H.R.= 1.46; 95% C.I.: 1.36–1.56), while the validation set had a 28% increased risk (H.R.= 1.28; 95% C.I.: 1.22–1.34) after adjustment for age or tumor stage. The PGS was effective even in subgroup analysis (epithelioid, biphasic and sarcomatoid tumors).

Another study that corroborates the prognostic value of nuclear grade and mitotic count in epithelioid MPM was published by Rosen et al. in 2018 [7]. They collected clinical and pathologic data of 776 patients diagnosed with epithelioid MPM between 1998 and 2014 from 17 institutions worldwide, involving many experts in mesothelioma pathology. Tumors were divided into three groups based on the mitotic count: low, 0−1 mitotic figures per 10 high power fields (/10 HPF); intermediate, 2−4/10 HPF; and high, ≥5/10 HPF. The low, intermediate and high groups were assigned mitotic count scores of 1, 2 and 3, respectively. Nuclear atypia was assessed in the area with the highest degree of atypia and was classified as mild, moderate and severe, which were given scores of 1, 2 and 3, respectively. A composite nuclear grade was assigned to each case by summing the individual scores for nuclear atypia and mitotic count. Nuclear grade I was accorded to tumors with a score of 2−3, grade II for scores 4−5, and grade III for score 6. Patients with nuclear grade III tumors had the worst median OS (8 months), followed by nuclear grade II (14 months) and nuclear grade I tumors (27 months, *p* < 0.0001). Necrosis was also evaluated and scored as present or absent. The authors revealed that the addition of necrosis to nuclear grade further stratified OS, allowing classification of epithelioid MPM into the following four distinct prognostic groups: (1) nuclear grade I tumors without necrosis (29 months); (2) nuclear grade I tumors with necrosis and grade II tumors without necrosis (16 months); (3) nuclear grade II tumors with necrosis (10 months) and (4) nuclear grade III tumors (8 months).

In light of these results and advances, a multidisciplinary group of pathologists, molecular biologists, surgeons, radiologists and oncologists, sponsored by EURACAN/IASCLC, was convened in 2018 to critically review the traditional WHO classification of MPM [9]. Nicholson et al. proposed an update of the 2015 classification system in order to include architectural patterns, along with stromal and cytologic features, that might refine treatment decision-making and avoid misdiagnosis. A crucial aspect was the proposal of a pathologic grading system for diffuse epithelioid pleural mesothelioma that would provide prognostic stratification. The authors recommended that the grading of epithelioid MPM should be routinely part of reporting for all types of samples, favoring a two-tier system of low and high grade, based on a combination of nuclear atypia, mitotic count, and the presence or absence of necrosis. The distinction between low- and high-grade neoplasm might improve patients’ risk stratification with potential therapeutic implications and would allow the identification of tumors with more aggressive behavior. Finally, for the same purpose, the aforementioned multidisciplinary group considered the addition of favorable and unfavorable histologic features, including architectural patterns, cytologic characteristics and stromal features.

In 2020, Zhang et al. conducted an independent external validation of this proposed two-tiered grading system in a large study involving 563 consecutive cases of epithelioid MPM, of which 87% of patients underwent biopsies only [8]. The two-tier nuclear grading separated tumors into low-grade and high-grade, showing a median OS of 19.3 months and 8.9 months, respectively. According to the three-tier nuclear grading system previously validated by Rosen et al. [7], the authors also divided tumor samples into three groups. Grade I epithelioid mesothelioma presented the most favorable median OS (24.7 months), followed by grades II (12.7 months) and III (7.2 months). On multivariate analysis, three-tier nuclear grade, two-tier nuclear grade and mitosis–necrosis (M–N) score were predictors of OS, independently of age, procedural type, solid-predominant growth pattern, necrosis and atypical mitosis (all *p* < 0.001 except two-tier nuclear grade, *p* = 0.001). Moreover, on the basis of their results, the authors also suggested sampling three sites or a maximum tissue dimension of at least 20 mm from a single site as optimal for nuclear grade assessment.

In the same year, Bilecz et al. proposed a comparative analysis of prognostic histopathologic parameters in subtypes of epithelioid pleural mesothelioma [17]. They investigated data of 192 patients diagnosed with epithelioid MPM between 1994 and 2015 from different European centers. Nuclear grading and M–N score were determined and correlated with clinicopathological parameters and OS. All tumor samples were retrospectively analyzed and collected into three groups according to the standard three-tier nuclear grading system. Patients with tumors of M–N scores 1, 2 and 3 had significantly different OS of 720 days, 386 days (*p* = 0.004) and 165 days (*p* = 0.0036), respectively. There was no significant difference in OS between nuclear grades 1 and 2. However, patients with nuclear grade 3 had significantly worse OS when compared to patients with nuclear grade 2 (median OS 123 versus 486 days, *p* = 0.0002). Histological subtypes were collapsed into three groups based on their overlapping survival curves. The tubulopapillary/microcystic group had a significantly longer OS than the solid/trabecular group (732 days versus 397 days, *p* = 0.0013). Conversely, pleomorphic tumors had the shortest OS (173 days). The solid/trabecular variants showed a significant association with both higher M-N scores (*p* < 0.0001) and higher nuclear grades (*p* = 0.007). On multivariate analysis, the M-N score was confirmed a robust and independent prognostic factor (*p* = 0.007). On one hand, this study corroborated the prognostic role of different architectural patterns of epithelioid MPM; on the other hand, it directly compared the prognostic impact of morphological growth pattern, the nuclear grade and the M–N score.

The same results were reported by Paajanen et al. in a cohort study comparing 43 tumors from long-survivor patients (more than 5 years OS) affected by epithelioid MPM, with 84 tumors from a reference group with average survival [18]. The aim was to investigate the prognostic impact of histopathologic and morphological features associated with extended survival in epithelioid MPM. Most of the tumors with better survival presented nuclear grade I (*n* = 34, 90%) and a tubulopapillary growth pattern (*n* = 30, 70%), while only one case showed necrosis. By contrast, nuclear grade II (*n* = 49, 61%) and solid subtype (*n* = 59, 70%) were observed more frequently in the reference cohort, also characterized by necrosis in 16 (19%) tumors. Statistical analysis found low nuclear grade (*p* < 0.001) and presence of exophytic polypoid growth (*p* = 0.024) were associated with prolonged survival.

More recently, a retrospective study conducted by Forest et al. assessed the association between molecular features and the prognostic stratification of epithelioid MPM, in addiction to tumor grading, cytological features, architectural and stromal patterns [19]. The authors collected data of 120 patients with a diagnosis of epithelioid MPM from 2000 to 2020. All diagnoses were reviewed and classified according to the 2015 WHO classification concerning epithelioid subtypes [2], while tumor grade was as defined in the EURACAN/IASCLC proposals [9]. Their results showed that high-grade tumors (HR = 3.09; 95% C.I.: 1.50–6.35, *p* = 0.002) and predominant tubular or tubulopapillary pattern (HR = 0.56; 95% C.I.: 0.32–0.99, *p* = 0.045) were related to OS. The study confirmed that tumor grading (*p* ≤ 0.001) had a major prognostic role in epithelioid MPM and revealed the adjunctive predictive value of the predominant architectural pattern (*p* = 0.001).

### 3.2. Diagnostic Pitfalls in Pleural Diffuse Mesothelioma

WDPM is a localized or multifocal tumor, exceptionally rare in the pleura, characterized by relatively indolent behavior [11]. Accordingly, the 2021 WHO classification has renamed it as WDPMT and merged into preinvasive mesothelial tumors to differentiate this type from diffuse mesothelioma [10]. Histologically, WDPMT is defined by papillary growth pattern, covered by a single layer of bland mesothelial cells without stromal invasion. Atypia and mitoses are absent.

Diagnosis is often challenging and requires an accurate histologic examination of the entire surgical specimen in order not to misdiagnose an underlying invasive diffuse mesothelioma with predominant surface papillary architecture. In this regard, biopsy samples should always include subpleural fat so that the extent of invasion can be assessed [9]. Moreover, correlation with imaging and surgical findings is always needed to exclude the presence of different subtypes of MPM.

WDPMT has intact BAP1 nuclear expression and no homozygous deletion of CDKN2A/p16; therefore, these features can be helpful in the differential diagnosis with diffuse MPM. However, Lee et al. reported BAP1 loss in unusual cases with synchronous or metachronous diffuse mesothelioma, corroborating the risk of diffuse papillary mesothelioma mimicking WDPMT [20]. PAX8 expression is also commonly seen in WDPMT, as demonstrated in the large case series of Sun et al. in 2019 [21]. Although PAX8 staining has been shown to be highly sensitive and specific for WDPMT compared with epithelioid MPM (*p* < 0.001), in which it is generally absent, an aberrant overexpression may be present between different histologic subtypes [21,22,23]. Reactive mesothelial proliferation represents another diagnostic pitfall; however, it is characterized by thinner papillae and copious associated inflammation.

As another part of the picture, morphology alone cannot distinguish between reactive process and the well-recognized new entity of MIS. The current definition refers to flat or slightly papillary single-layer relatively bland mesothelial cells growing along the pleural surface, which have lost BAP1 or MTAP by IHC and/or the homozygous deletion of CDKN2A/p16 by FISH [10]. Churg et al. firstly described the cases of MIS with flat or slightly papillary single-layer surface mesothelial proliferation with BAP1 loss and/or CDKN2A homozygous deletion in patients with recurrent non-resolving pleural effusions and without evidence of tumor on imaging or thoracoscopy [13,14]. The authors reported 11 cases of MIS (9 pleural and 2 peritoneal), with no evidence of invasive mesothelioma developing for at least 1 year. In the larger series of 10 patients, 7 developed invasive MPM 12 to 92 months after biopsy, with 3 patients still free of invasive disease at 12, 57, and 120 months, respectively [13].

Diagnosis may be challenging, given the need for a multidisciplinary correlation among clinical, histologic, immunohistochemical and/or molecular, and radiologic findings. By definition, there must be no evidence of a tumor on imaging or by direct visualization of pleura cavity, and invasive mesothelioma must be absent, to formulate a diagnosis of MIS. Furthermore, pleural fluid cytology specimens are not adequate samples; the tissue biopsies are effectively needed to rule out subpleural fat invasion. It is worth emphasizing that morphology alone is insufficient for a diagnosis of MIS, while demonstration of BAP1 loss by IHC or CDKN2A/p16 homozygous deletion by FISH are required [13,24]. Besides, loss of MTAP expression by IHC occurs in nearly 90% of tumors with homozygous deletion of CDKN2A/p16, because these two genes reside close together within the same chromosome region [25]. Therefore, MTAP IHC can be used as a surrogate marker for CDKN2A FISH assay [26,27].

Another major issue concerns molecular heterogeneity in MPM. It has recently been demonstrated that BAP1 alterations represent a very early event in the development of a subset of mesotheliomas; however, to date, the evidence supports that BAP1 and CDKN2A loss occur in up to 70% of cases, and therefore the diagnosis of MPM cannot certainly be excluded in the absence of these molecular abnormalities [28]. In addition, although BAP1 loss and CDKN2A/p16 homozygous deletion have emerged as reliable markers for the differential diagnosis of benign mesothelial proliferation versus mesothelioma, they were revealed not to be appropriate for distinguishing mesothelioma from other malignant tumors [29,30,31,32,33,34,35,36]. Sensitivity and specificity of different molecular markers for diagnosis of pleural mesothelioma are shown in Table 3.

## 4. Discussion

Malignant mesothelioma has been misdiagnosed for decades, because of its rareness and high mortality. After several years of research and controversies, diagnosis modalities and treatment strategies of MPM are still far from being standardized. Despite biological and molecular experimentation and innovation, the pathologic classification of MPM has been traditionally limited to three histologic subtypes identified by the WHO in 2015 [2]. The failure of systemic and locoregional therapies for both epithelioid and non-epithelioid classes has led to the requirement of a deeper understanding of MPM and an exhaustive pathologic classification considering the latest molecular breakthroughs.

Historically, the main histologic types of MPM have been the major predictors of survival [37,38]. Epithelioid histology is a strong prognostic factor, related to a better prognosis compared with biphasic and sarcomatoid subtypes [2]. Although the predictive value of nuclear grading in different types of cancer is already well established, such as breast, renal cell and bladder carcinoma, grading has never been recommended before for epithelioid MPM, given its poor prognosis [39]. Nevertheless, a renewed interest in proposing an exhaustive grading system for epithelioid MPM has emerged from the scientific community over the past decade [3]. Various features have been evaluated; at last, nuclear atypia, mitotic count and necrosis have proved as independent prognostic factors [5,6,7,8].

In this scenario, in July 2018, the histologic classification of MPM was reviewed by a multidisciplinary group convened by the EURACAN/IASLC [9]. In contrast with the traditional three-tier nuclear grade stratification [7], they proposed a two-tier system of low and high grade for epithelioid diffuse pleural mesothelioma, based on a combination of nuclear features, mitotic rate, and the presence or absence of necrosis [9]. Tumors should be graded based on the highest-grade features [5,7,9]. On the thrust of this proposal and with the goal of a more accurate risk stratification of patients, the 2021 WHO Classification of Tumors of the Pleura adopted the EURACAN/IASLC grading system. All nuclear grade 1 tumors (with or without necrosis) and nuclear grade 2 tumors without necrosis have been reclassified as low-grade; nuclear grade 2 tumors with necrosis and any nuclear grade 3 tumors have been reclassified as high-grade [10].

As a matter of fact, grading and subtyping of epithelioid MPM represents the most significant updates of the latest WHO classification and have proved major independent predictors of survival [24,39,40]. Their prognostic significance might have very important clinical implications, particularly considering the extensive morphologic heterogeneity of epithelioid MPM. To date, consensus has been obtained that grading should be limited to epithelioid MPM, because patients with epithelioid histology, showing a better prognosis, would benefit the most from improved risk stratification. This novel grading system should be applied to resection specimens as well as biopsies, although it cannot yet be extended to biphasic and sarcomatoid subtypes, due to their still ominous prognosis [9,10].

Several recent studies highlighted that recognition of different growth patterns is of paramount importance since distinct subtypes have prognostic value [17,18,19,24,40]. Even though architectural patterns were discussed in the 2015 WHO classification, architectural patterns, cytologic features and stromal characteristics have not been formally incorporated until the last 2021 classification. Histologic features present in epithelioid diffuse mesothelioma that are associated with better prognosis consist of tubulopapillary, trabecular, or adenomatoid architectural patterns, lymphohistiocytoid cytologic features, or the presence of myxoid stroma [17,18,41]. Conversely, unfavorable histologic features include micropapillary patterns, solid patterns (when present in more than or equal to 50% of a tumor), rhabdoid or pleomorphic cytologic features, or the presence of necrosis [4,7,17,18,42]. These patterns should be reported in percentages in the resected specimens and mentioned in the report of smaller samples, such as biopsies.

Whereas MPM with pleomorphic and lymphohistiocytoid features were classified under epithelioid tumors in the 2015 WHO classification [2], the 2021 pathologic update has redefined these architectural patterns as cytological features and reclassified mesotheliomas with these characteristics as epithelioid, biphasic or sarcomatoid on the basis of coexistent tumor cell morphology [10]. Similarly, transitional cytologic features represent another novelty of the latest classification that has upgraded this morphology into the sarcomatoid class, given recent evidence revealing the presence of transitional features to be associated with worse prognosis [24]. However, as for the grading, for the histological characteristics the prognostic significance is also very limited in the context of sarcomatoid MPM in relation to their adverse prognosis.

The correct identification of this mixture of architectural patterns and cytologic and stromal features in epithelioid diffuse mesothelioma is crucial for several reasons. First of all, their prognostic value with subsequent improved patient risk stratification would have therapeutic implications and would be a landmark for treatment allocation in the setting of a multimodal strategy. Secondly, it is important to be aware of the morphological heterogeneity of epithelioid mesothelioma in order to perform adequate IHC workup and to avoid misdiagnosis due to histologic similarity with other tumor types.

The introduction in the 2021 WHO classification of two distinct entities—WDPMT and MIS—within the group of benign and preinvasive mesothelial tumors, has increased the already non-negligible diagnostic challenges. Since the prognosis of WDPMT is very good, with occasional local recurrences, differentiation from diffuse epithelioid MPM with predominant papillary pattern is crucial [40]. BAP1 IHC and CDKN2A/p16 FISH may be helpful in identifying WDPMT, and the diagnosis of WDPMT should be questioned when BAP1 expression is lost by IHC or homozygous deletion in CDKN2A is detected by p16 FISH [40].

Similarly, it is worth emphasizing that morphology alone is insufficient for diagnosis of MIS, and demonstration of BAP1 loss by IHC or CDKN2A homozygous deletion by FISH is required for diagnosis [13,24]. How long MIS can persist without evolving into an invasive disease is unclear, although Churg et al. reported a progression 12 to 92 (median: 60) months after a biopsy diagnosis of MIS [13]. The concept of precancerous lesion for MIS should always be kept in mind: the management of these patients should be discussed at multidisciplinary tumor board as there is usually a long latency period before the development of an invasive mesothelioma. However, after a median follow up of 5 years, up to 70% of MIS will progress into invasive MPM [13].

The assessment of lack of invasiveness is one more major diagnostic pitfall in the differential diagnosis between preinvasive tumors, such as WDPMT and MIS, from diffuse mesothelioma. It is mandatory that ideal biopsy samples should include subpleural fat, so that the extent of invasion can be assessed. This is fundamental to distinguish well-differentiated superficial mesotheliomas and to not misdiagnose deeper areas of invasive growth.

There is also a need to refine the number and location, as well as the depth, of surgical samples, to ensure the presence of sufficient material to make an accurate diagnosis and recognize any different histologic subtypes. The EURACAN/IASLC expert consensus proposal consists of the sampling of at least three separate areas from the pleural cavity, with subpleural fat, including any area of interest identified on pre-surgical imaging [9]. In fact, a higher number of biopsies have been shown to provide better concordance with tumor subtype, and better accord has been found between thoracoscopic biopsy samples rather than needle biopsy specimens [43].

Thoracoscopic biopsies are the gold standard for suspected MPM [44], even if other biopsy methods are less invasive and may be more appropriate in selected cases. Besides providing a pathological diagnosis with a sensitivity of 95% and specificity of 100%, video-assisted thoracic surgery (VATS) has a potentially therapeutic purpose, allowing evacuation of pleural effusion and talc pleurodesis, as well as offering the additional advantage of staging assessment of visceral and parietal pleura invasion [45]. Furthermore, it is important to underline that VATS can be performed not only under general anesthesia but also under local anesthesia on nonintubated patients, equally becoming a suitable choice for frail patients [46]. Unfortunately, whenever a locally advanced disease prevents VATS from being performed, due to the presence of a totally obliterated pleural space, an open pleural biopsy realized through a limited muscle-sparing incision within an intercostal space is a valid surgical choice [45]. Effective options for patients clinically unfit for surgery are ultrasound- and computed-tomography-guided pleural biopsy, even if medical thoracoscopy has been shown to have higher diagnostic sensitivity than image-guided techniques, with a low complication rate [47].

Reactive mesothelial proliferation represents another diagnostic trap. The IHC analysis of BAP1 loss clearly has value in confirming MPM diagnosis in atypical mesothelial proliferations and it is recommended as an effective diagnostic tool, although the application of the antibody is not yet standardized and diagnosis of MPM cannot certainly be excluded in case of retained expression of BAP1 [16,28].

## 5. Conclusions

This review highlighted major advances in the latest WHO Classification of Pleural Tumors, which have led to improvements in the diagnosis of these neoplasms and revealed not a merely speculative and clinical significance, but a potential therapeutic role.

Morphological heterogeneity of epithelioid mesothelioma and the presence of preinvasive tumors should be always considered to avoid misdiagnosis.

Despite diagnostic challenges, the recognition of MIS may provide chances for early intervention in this fatal disease.

Pathologic update of pleural mesothelioma classification, with the acknowledgement of the prognostic value of grading and subtyping of epithelioid MPM, may improve patient risk stratification and have pivotal future therapeutic implications.

## Figures and Tables

**Table 1:** Major changes in 2021 WHO Classification of Tumors of the Pleura.

2015 Classification	2021 Classification	
Well-differentiated papillary mesothelioma (WDPM)	Pre-invasive mesothelial tumors	
	Well-differentiated papillary mesothelial tumor (WDPMT) Papillary formations covered by a single layer of bland mesothelial cells without stromal invasion Mesothelioma in situ A single layer of relatively bland mesothelial cells growing along the pleural surface with loss of BAP1 or MTAP by IHC * or homozygous deletion of CDKN2A by FISH ^#^ Adenomatoid tumor	
Malignant Mesothelioma	Diffuse Mesothelioma	
Three main histologic subtypes (epithelioid, biphasic, and sarcomatoid)	Architectural patterns, cytologic and stromal features are more formally incorporated, as histologic prognostic factors, for epithelioid diffuse mesothelioma	
	Favorable features - tubulopapillary, trabecular, or adenomatoid architectural patterns - lymphohistiocytoid cytologic features - presence of myxoid stroma	Unfavorable features - micropapillary pattern, solid pattern (≥50%) - rhabdoid or pleomorphic cytologic features - presence of necrosis
	Nuclear Grading for epithelioid diffuse mesothelioma Introduction of a two-tier nuclear grading system that incorporates nuclear atypia, mitoses, and the presence or absence of necrosis.	
Nuclear Grade 1—with/without necrosis Nuclear Grade 2—without necrosis	Low grade	
Nuclear Grade 2—with necrosis Nuclear Grade 3	High grade	
Histological Differential Diagnosis	BAP1, EZH2, MTAP loss by IHC * or CDKN2A homozygous deletion by FISH ^#^ are introduced as markers for differential diagnosis of benign mesothelial proliferation versus mesothelioma.	
Diagnosis of biphasic mesothelioma with a minimum of 10% of either epithelioid or sarcomatoid Component	Diagnosis of biphasic mesothelioma is unchanged in definitive resection specimens, but a minimum of 10% of either epithelioid or sarcomatoid component is no longer required in smaller specimens (biopsy and cytologic samples)	

* IHC: immunohistochemistry; ^#^ FISH: fluorescence in situ hybridization.

**Table 2:** Histological prognostic factors for pleural diffuse epithelioid mesothelioma.

Authors	Study Design	N. of Patients	Research Parameters	Prognostic Factors
Kadota et al. [5] 2012	Retrospective	232	Nuclear atypia	*p* = 0.012
			Nuclear/cytoplasmic ratio	/
			Chromatin pattern	/
			Intranuclear inclusions	/
			Prominence of nucleoli	/
			Mitotic count	*p* < 0.001
			Atypical mitoses	/
			Nuclear grade	*p* < 0.001
Pelosi et al. [6] 2018	Retrospective	940	Histological subtypes	/
			Necrosis	/
			Mitotic count	/
			Ki-67 labeling index	/
			Pathologic Grading System (PGS)	*p* < 0.01
Rosen et al. [7] 2018	Retrospective Multicentric	776	Mitotic count	*p* = 0.001
			Nuclear atypia	*p* = 0.009
			Nuclear grade	*p* < 0.0001
			Mitosis–Necrosis score	*p* < 0.0001
Zhang et al. [8] 2020	Retrospective	563	Mitosis–Necrosis score	*p* < 0.001
			Three-tier nuclear grade	*p* < 0.001
			Two-tier nuclear grade	*p* = 0.001
Bilecz et al. [17] 2020	Multicentric Cohort	192	Histological subtypes	/
			Nuclear grade	/
			Mitosis–Necrosis score	*p* = 0.007
Paajanen et al. [18] 2020	Cohort	1010	Growth pattern	*p* = 0.024
			Histological subtypes	/
			Nuclear grade	*p* < 0.001
			Necrosis	/
Forest et al. [19] 2021	Retrospective	120	Tumor grading	*p* < 0.001
			Tumor/stroma ratio	/
			Myxoid stroma	/
			Tertiary lymphoid structures	/
			Predominant architectural pattern	*p* = 0.001

**Table 3:** Sensitivity and specificity of molecular markers for differential diagnosis of pleural mesothelioma.

Authors	N. of Patients	Molecular Markers	Diagnostic Technique	Sensitivity	Specificity
Lee et al. [20] 2018	8	BAP1 (WDPM *)	IHC ^#^	/	/
Sun et al. [21] 2019	75	PAX8 (WDPM *)	IHC ^#^	94%	88%
Illei et al. [25] 2003	31	CDKN2A (MPM ^§^)	Dual-color FISH ^$^	/	/
Berg et al. [26] 2018	20	MTAP (MPM ^§^)	IHC ^#^	65%	/
Hwang et al. [32] 2016	20	CDKN2A BAP1	FISH ^$^	80% 15%	/
Monaco et al. [33] 2015	154	GLUT-1	IHC ^#^	20%	100%
Yoshimura et al. [36] 2019	67	EZH2, BAP1 MTAP 9p21	IHC ^#^/FISH ^$^	44.7% 52.6% 47.4% 65.8%	100% 100% 100% 100%

* WDPM: well-differentiated papillary mesothelioma; ^#^ IHC: immunohistochemistry; ^§^ MPM: malignant pleural mesothelioma; ^$^ FISH: fluorescence in situ hybridization.

## Data Availability

The data presented in this study are available on request from the corresponding author.
